# Chinese bayberry (*Myrica rubra* Sieb. et Zucc.) leaves proanthocyanidins inhibit intestinal glucose transport in human Caco-2 cells

**DOI:** 10.3389/fphar.2024.1284268

**Published:** 2024-03-11

**Authors:** Mengting Wang, Haiguang Mao, Zhijian Ke, Jianchu Chen, Lili Qi, Jinbo Wang

**Affiliations:** ^1^ School of Biological and Chemical Engineering, NingboTech University, Ningbo, China; ^2^ College of Biosystems Engineering and Food Science, Zhejiang University, Hangzhou, China

**Keywords:** proanthocyanidins, glucose transport, human Caco-2 cells, GLUT2, SGLT1

## Abstract

**Background:** The hypoglycemic effects of Chinese bayberry leaves proanthocyanidins (BLPs) have been demonstrated. It is unclear, nevertheless, whether BLPs reduced postprandial blood glucose levels by regulating glucose uptake and glucose transport.

**Method:** This study investigated the effect of BLPs (25, 50, and 100 μg/mL) on glucose uptake and glucose transport in human intestinal epithelial cells (Caco-2 cells). The uptake of 2-Deoxy-2-[(7-nitro-2,1,3-benzoxadiazol-4-yl) amino]-D-glucose (2-NBDG) and disaccharidases activity in Caco-2 cells were measured. The glucose transport ability across the cell membrane was determined using the established Caco-2 monolayer model. The transcript and protein levels of key glucose transporters were analyzed using real-time quantitative polymerase chain reaction (RT-qPCR) and western blotting, respectively.

**Results:** The results showed that BLPs significantly decreased glucose uptake and disaccharidases activity (*p* < 0.05). Otherwise, BLPs treatment obviously inhibited glucose transport across the Caco-2 monolayer in both simulated-fast (5 mM glucose) and simulated-fed (25 mM glucose) conditions. It was attributed to the suppression of glucose transporter2 (GLUT2) and sodium-dependent glucose cotransporter 1 (SGLT1) by BLPs. BLPs were found to significantly downregulated the transcript level and protein expression of glucose transporters (*p* < 0.05). Meanwhile, the mRNA expression of phospholipase C (PLC) and protein kinase C (PKC) involved in the signaling pathway associated with glucose transport were decreased by BLPs.

**Conclusion:** These results suggested that BLPs inhibited intestinal glucose transport via inhibiting the expression of glucose transporters. It indicated that BLPs could be potentially used as a functional food in the diet to modulate postprandial hyperglycemia.

## 1 Introduction

Type 2 diabetes is a metabolic disorder with hallmarks of insulin resistance and hyperglycemia, closely related to other chronic diseases (Guo et al., 2023). The main trigger is excess glucose accumulation originating from the carbohydrate-rich diet. In general, ingested carbohydrates are hydrolysed to monosaccharides, mainly glucose, absorbed into intestinal epithelial cells and transported to the circulation, ultimately causing a postprandial blood glucose response (Wu et al., 2020). During the process of carbohydrate digestion, glucose production is facilitated by digestive enzymes such as amylase, glucosidase, sucrose and maltase (Barber et al., 2022). Glucose is then incorporated through the small intestine *via* the two main glucose transporters, sodium-dependent glucose cotransporter 1 (SGLT1) and glucose transporter 2 (GLUT2). Therefore, the inhibition of hydrolysis enzymes and glucose transport are two effective ways to control postprandial blood glucose levels (Sun and Miao, 2020).

Our previous research has identified the hypoglycemic potential of Chinese bayberry leaves proanthocyanidins (BLPs), a novel functional component with abundant biological benefits. The inhibitory effect of BLPs on α-amylase and α-glucosidase activity has been reported, which is conducive to retarding starch digestibility (M. Wang, et al., 2022a) (M. Wang, et al., 2022b). It has also been confirmed that BLPs were transported across the Caco-2 monolayer (Tao et al., 2020). However, whether BLPs can regulate glucose uptake and transport has yet to be explored.

Recently, several phytochemicals have been found to effectively reduce glycaemic levels by targeting glucose transport proteins *in vitro* and *in vivo* (Pico and Martínez, 2019; Abioye et al., 2022). For example, apple polyphenol extracts were reported to reduce postprandial blood glucose levels in mice and humans by inhibiting the intestinal SGLT1 (Schulze et al., 2014). Gallic acid and its derivatives exerted a glucose transport inhibition effect in Caco-2 cell monolayers. It was confirmed that this effect was dependent on GLUT2-specific inhibitors (H. Wang et al., 2021). In another study, bee pollen extract from *Camellia sinensis* L. was found to inhibit glucose uptake and glucose transport across Caco-2 cell monolayers by directly interacting with glucose transporters *via* competition with glucose (Q. Li et al., 2021). However, the specific molecular mechanisms of these phenolic compounds on glucose transporters are inconsistent and remain to be unraveled. Previously, some procyanidins have been reported to control postprandial hyperglycemia by inhibiting glucose uptake and glucose transport, such as peanut skin procyanidins (Tamura et al., 2015) (M. Liu et al., 2023), A-type procyanidins from litchi pericarp (X. Li et al., 2016) and procyanidins derivatives in cinnamon free phenolic extract (Y. Liu et al., 2023). These findings suggested that the suppressive effect of procyanidins on glucose transport was related to their structural characteristics, such as linkage type and degree of polymerization.

BLPs have been characterized as a category of prodelphinidins with terminal and extension units of epigallocatechin gallate (EGCG), which distinguishes them from the subunits of procyanidins (M. Wang, et al., 2022a). It is still unknown whether BLP, as a new category of proanthocyanidins, can play a regulatory role in glucose transport. Therefore, the effect of BLPs on glucose absorption and glucose transport was investigated in this study. The Caco-2 cell monolayer model is commonly used to mimic the human small intestinal epithelial cells including endogenous transport proteins and brush border disaccharidases (Pico et al., 2019). In this study, we established a Caco-2 cell monolayer model, and estimated the glucose transmembrane transport capacity under simulated-fast (5 mM glucose) and simulated-fed (25 mM glucose) conditions. The transcription and protein expression levels of glucose transporters (GLUT2 and SGLT1) were determined by qPCR and Western blot analysis. This study aimed to provide new insights into the molecular mechanisms of the hypoglycemic effect of BLPs.

## 2 Materials and methods

### 2.1 Materials

Proanthocyanidins from Chinese bayberry (*Myrica rubra Sieb.* et Zucc.) leaves (BLPs) were prepared following our previous methods (M. Wang et al., 2022b). The proanthocyanidin content of BLPs was 85.7% ± 1.1% (EGCG equivalent) and the mean degree of polymerization of BLPs was 7.30. Additional structural information can be seen inSupplementary Material S1. The human Caco-2 cell line was obtained from the Cell Bank of the Shanghai Institute of Cell Biology, Chinese Academy of Sciences (Shanghai, China).

### 2.2 Chemicals

Guar gum and fluorescein sodium were purchased from Aladdin (Shanghai, China). The glucose assay kit (GOPOD format) was purchased from Megazyme (Wicklow, Ireland). Dulbecco’s modified eagle medium (DMEM) and Methylthiazolyldiphenyl-tetrazolium bromide (MTT) were purchased from Sigma-Aldrich (St, Louis, USA). Fetal bovine serum (FBS), penicillin-streptomycin, and trypsin-EDTA (0.25%) were purchased from Gibco, Life Technologies (Grand Island, NY, USA). Phloretin (PT) and phlorizin (PZ) were purchased from Yuanye Bio-Tech (Shanghai, China). 2-Deoxy-2-[(7-nitro-2,1,3-benzoxadiazol-4-yl) amino]-D-glucose (2-NBDG) was obtained from Maokang Biotechnology (Shanghai, China). TRIGene reagent was purchased from GenStar (Beijing, China). PrimeScript™ RT reagent kit with gDNA eraser (Perfect Real Time) was purchased from Takara (Tokyo, Japan). Power SYBR green master mix was purchased from Applied Biosystems, Thermo Fisher Scientific (Wilmington, DE, United States). SDS-PAGE loading buffer (2×), PBS, BCA protein assay kit, and enhanced chemiluminescence (ECL) detection assay were purchased from Beyotime Biotechnology (Shanghai, China). The antibodies including β-actin, GLUT2 and SGLT1 were purchased from Abcam (Cambridge, United Kindom) and Cell Signaling Technology (Danvers, Massachusetts, United States). ExpressPlus PAGE Gels, Tris-MOPS-SDS buffer, β-actin and the secondary antibodies were purchased from GenScript Biotech (Nanjing, China).

### 2.3 Glucose diffusion and absorption determination

According to the previous report (Zheng et al., 2021), a mixture of 25 mL of glucose solution (100 mM) and BLPs (0–1 mg/mL) or guar gum (1 mg/mL) was dialyzed in a dialysis bag (10,000 MWCO) against 225 mL of deionized water at 37°C. The glucose content in the dialysates was determined at 30, 60, 90, 120, 180, and 300 min, respectively. Meanwhile, 1 mg/mL of BLPs or guar gum was added to 25 mL of glucose solution (5, 10, 50, and 100 mM), and dialysis was performed for 300 min. In the end, the glucose content in the dialysates was determined.

### 2.4 Cell culture

Human Caco-2 cells were cultured in a complete medium consisting of DMEM supplemented with 20% FBS and 1% penicillin-streptomycin. The cells were maintained at 37°C in an atmosphere of 5% CO_2_. Trypsin was used to dissociate the cells for cell passage or experiments.

### 2.5 Cell viability assay

The effect of BLPs on Caco-2 cell viability was evaluated using the MTT method (Zein et al., 2020; Teng et al., 2022). In brief, digested cells (1 × 10^4^ cells/well) were pre-cultured until reaching 80% confluence, and then co-incubated with 100 μL of BLPs (concentration ranged from 20 to 250 μg/mL) for 24 h. After being washed thrice, the cells were treated with 100 μL of MTT solution for 4 h and 200 μL of DMSO solution for 10 min. The absorbance at 550 nm was measured. The cell viability of cells in the control group was regarded as 100%.

### 2.6 Glucose uptake assay

The glucose uptake assay was conducted according to the previous report (Zakłos-Szyda et al., 2019) with some modifications. In brief, the digested cells (1 × 10^4^ cells/well) were seeded in a 96-well plate and cultured for 14 days to form a cell monolayer. Afterwards, the cells were treated with 100 μL of BLPs (concentration ranged from 20 to 100 μg/mL) for 24 h, followed by treatment with 100 μL of 2-NBDG (100 μM) at 37°C for 30 min. The fluorescence value was measured at Ex/Em = 485/535 nm and Ex = 485 nm, and the glucose uptake of cells in the control group was regarded as 100%.

### 2.7 Maltase and sucrase activity determination

The maltase and sucrase of Caco-2 cells in the presence of BLPs were determined according to the previous study with slight modifications (Q. Li et al., 2020). The digested cells (4 × 10^4^ cells/well) were seeded in a 24-well plate and cultured for 14 days. After being washed thrice, the cells were treated with 800 μL of the substrate (7 mM maltose or 28 mM sucrose) and 200 μL of BLPs (25, 50 and 100 μg/mL) at 37°C for 2 h. The BLPs were replaced by PBS as a negative control and replaced by the acarbose (50 μg/mL) as a positive control. The glucose content in the culture supernatant was measured (Teng, et al., 2023) and calibrated with protein concentration, and then the enzyme activity inhibition rate was calculated.

### 2.8 Caco-2 cell monolayer glucose transport assay

**FIGURE 1:**
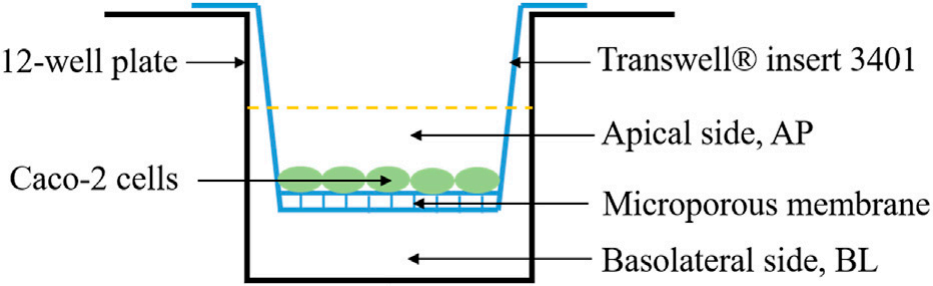
Schematic diagram of Caco-2 cell monolayer model.

The Caco-2 cell monolayer was established following the method of Chen et al. (Chen et al., 2019) with slight modifications. The digested cells (5 × 10^4^ cells/well) were seeded in a 12-well transwell insert. The culture medium on the apical (AP) side and basolateral (BL) side was 0.5 mL and 1.5 mL, respectively (Figure 1). After culture of 21 days, transepithelial electrical resistance (TEER) values and sodium fluorescein transmittance of the Caco-2 cell monolayer were measured.

The glucose transport assay was conducted under low-glucose (5 mM) and high-glucose (25 mM) conditions. The medium on the AP side was replaced with DMEM containing glucose and BLPs at 37°C for 120 min, together with PBS buffer on the BL side. At this period, the glucose content on the BL side was determined at 30, 60, 90, and 120 min, respectively. The percentage of glucose transport in the control group was regarded as 100%. Phloretin and phlorizin (2 mM) were set as positive controls in this study.

### 2.9 Real-time quantitative PCR analysis

The total RNA was extracted from Caco-2 cells treated with BLPs using TRIGene reagent, and then reverse transcribed into a cDNA template using PrimeScript™ RT reagent kit with gDNA eraser. The mRNA expression of genes was detected by RT-qPCR analysis using SYBR Green Mix, cDNA and primers in a Quant Studio 3 Real-Time PCR System (Applied Biosystems, United States). The expression of *β-actin* was used as an internal control and the transcription level was calculated using the 2^−ΔΔCT^ equation. The primer sequences of mRNA are listed in Table 1.

**TABLE 1:** Primer sequences of mRNA for RT-qPCR.

mRNA	Forward primer sequences (5′-3′)	Reverse primer sequences (5′-3′)
*β-actin*	TCA​TGA​AGT​GTG​ACG​TGG​ACA​TC	CAG​GAG​GAG​CAA​TGA​TCT​TGA​TCT
*GLUT2*	AGG​CAG​GGC​GAC​GTT​CTC​TC	CAG​CAG​CAC​AAG​TCC​CAC​TGA​C
*SGLT1*	CAT​CTA​CGC​CAA​GGT​CCG​CAA​G	TGC​CCA​CTT​TGT​GCT​GAC​TGC
*PKC*	TGA​GGA​GGA​TCG​AAT​GAG​AAG	CAG​GAC​AGG​ATG​GCA​AGG​A
*PKA*	CCA​TCA​AGG​CTA​TAT​CCA​GGT​C	TGC​CTT​ATT​GTA​GCC​CTT​GC
*PLC*	GGC​GAG​GTG​TCA​GTG​AAT​GG	CAG​GTC​TCC​TTT​GAA​TCC​ATC​TC

### 2.10 Western blot analysis

The total protein was extracted from Caco-2 cells treated with BLPs using lysis buffer and then denatured by boiling in SDS-PAGE loading buffer for 5 min. Equal amounts of protein were resolved on a 10% SDS-PAGE gel under 150 V and transferred to a polyvinylidene fluoride (PVDF) membrane (0.45 μm). The membranes were then blocked and incubated separately with GLUT2 and SGLT1 antibodies, followed by incubation with HRP-conjugated secondary antibodies. Western blots were performed with ECL reagent using a chemical luminescence imaging analysis system (Tanon, China).

### 2.11 Statistical analysis

All data were presented as means ± standard deviation (means ± SD.) of three independent experiments. Multiple comparisons between groups were performed variance analysis using one-way ANOVA in IBM SPSS Statistics 20 software, and significant difference analysis between groups was performed using Duncan’s test (*p* < 0.05).

## 3 Results and discussion

### 3.1 BLPs retard glucose diffusion and show adsorption capacity for glucose

**FIGURE 2:**
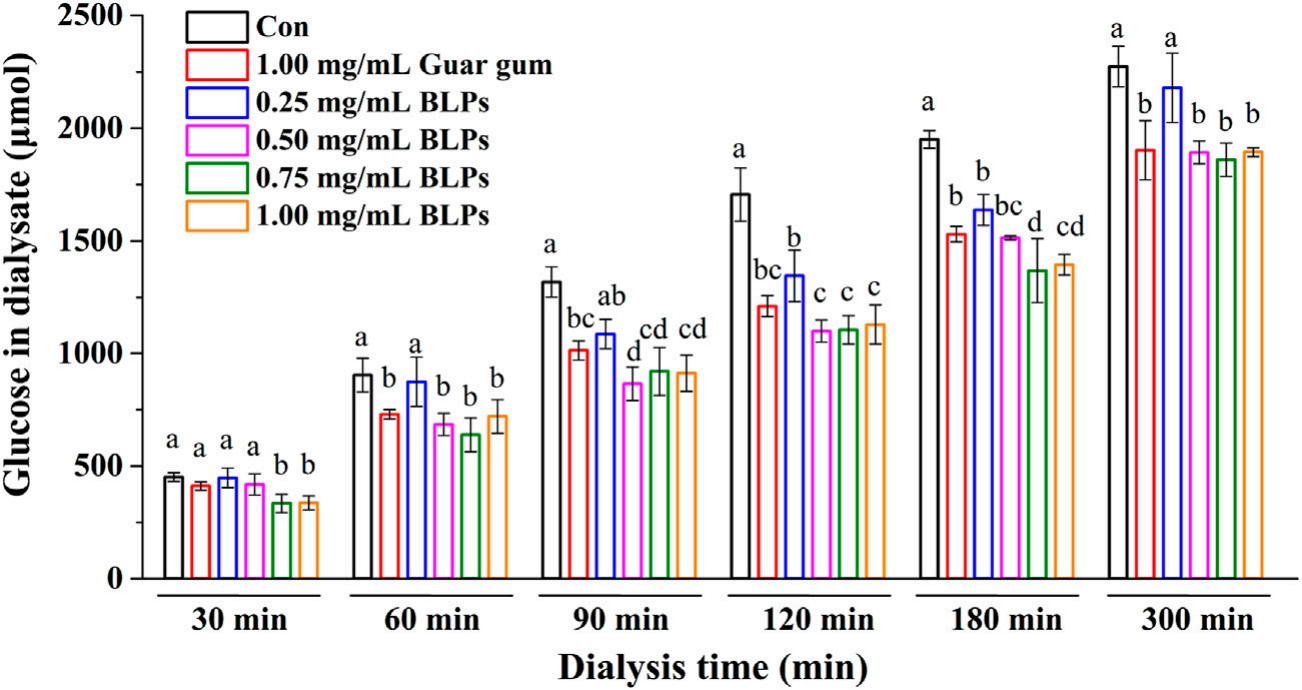
Effect of BLPs on the glucose in the dialysate. Different letters at each time represent significant differences (*p* < 0.05). The data at different times in the same group had significant differences (*p* < 0.05).

The effects of BLPs on glucose diffusion and glucose adsorption were determined by the glucose dialysis method. As seen in Figure 2, the glucose content in the dialysates increased in a time-dependent manner, indicating a glucose diffusion movement. Compared with the control group, BLPs and guar gum effectively reduced the glucose diffusion rate, and the reduction was equivalent when their addition amount was 1.00 mg/mL. This phenomenon is consistent with other botanical extracts that slowed glucose diffusion due to their absorption capacity to glucose. For example, agrimony extract (50 g/L) and avocado extract (50 g/L) decreased the overall glucose movement by 71% and 60%, respectively, compared to the control (Gallagher et al., 2003); fractions from pomelo fruitlets (10 mg/mL) significantly decreased the glucose diffusion velocities from 0.172 to 0.087–0.097 mg/(mL·h) (H. Liu et al., 2021); two extracts from Cladodes Flour exhibited a glucose diffusion velocities reduction of 35.2% and 37.9% (Nuñez-López et al., 2013).

When dialysis reaches equilibrium (300 min), the difference in diffused glucose content between BLPs (or guar gum) and the control is considered as glucose adsorption capacity (Ou et al., 2001). According to the results listed in Table 2, BLPs had a comparative glucose adsorption capacity with guar gum, suggesting that BLPs might affect the absorption of glucose in the small intestine. However, the dialysis method cannot simulate the dynamic process of glucose absorption in the small intestine directly. Therefore, the effect of BLPs on glucose absorption and transport will be further explored in human Caco-2 cells in the following experiments.

**TABLE 2:** Effect of BLPs on the glucose adsorption capability.

	Glucose adsorption capacity (mmol/g)			
	100 mM glucose	50 mM glucose (b)	10 mM glucose	5 mM glucose
1.00 mg/mL BLPs	16.15 ± 1.71^a^	8.67 ± 3.05	2.47 ± 0.40^c^	1.12 ± 0.26^c^
1.00 mg/mL guar gum	16.80 ± 1.56^a^	8.09 ± 0.69	2.71 ± 0.49^c^	1.44 ± 0.46^c^

Note: different letters in the same line represented significant differences (*p*< 0.05).

### 3.2 BLPs inhibit glucose uptake in Caco-2 cells

**FIGURE 3:**
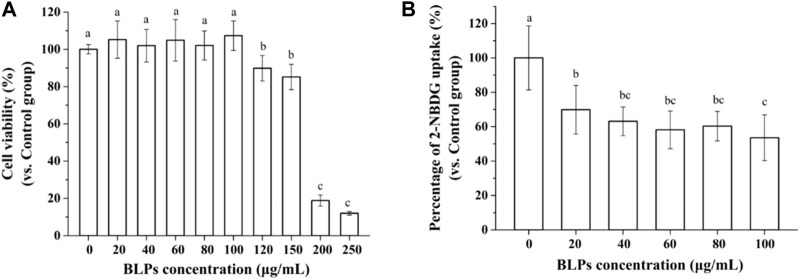
Effect of BLPs on the viability of Caco-2 cells **(A)**, and 2-NBDG uptake in Caco-2 cells **(B)**. Data are expressed as mean ± S.D. (*n* = 6). Different letters in the figure represent significant differences (*p* < 0.05).

In this study, it was found that concentrations of BLPs below 100 μg/mL did not significantly affect the viability of Caco-2 cells, which was considered not cytotoxic to cells (Figure 3A). The effect of BLPs on the glucose uptake in Caco-2 cells was also investigated. As shown in Figures 3B, 20–100 μg/mL of BLPs significantly decreased the 2-NBDG uptake of Caco-2 cells by 30.15%–46.46% compared with the control group. As previously reported in the literature, incubation with phenolic compounds from *Viburnum opulus* fruit reduced glucose uptake about by 7%–19% in comparison with the control group (Zakłos-Szyda et al., 2019). Additionally, Korean red ginseng fraction at concentration of 200–500 μg/mL caused a significant reduction in glucose uptake in Caco-2 cells by 29.2%–55.1% (Park et al., 2020). Similarly, black currants anthocyanins (0.66–6.6 μg/mL) inhibited glucose uptake by 56.1%–69.1% (Barik et al., 2020).

### 3.3 BLPs inhibit sucrase and maltase activity in Caco-2 cells

Sucrase and maltase activities were determined in Caco-2 cells treated with BLPs or acarbose (Table 3). As expected, BLPs inhibited the Caco-2 disaccharidases activity, illustrating the potential to slow the digestion of carbohydrate-rich foods. Our previous study demonstrated that BLPs had a strong inhibitory effect on α-amylase and α-glucosidase *in vitro* and *in vivo*. These findings indicated the hypoglycemic potential of BLPs.

**TABLE 3:** Effect of BLPs on the sucrase and maltase activity.

Samples	Inhibition%	
	Sucrase activity	Maltase activity
50 μg/mL acarbose	71.00 ± 9.98^a^	54.22 ± 4.18^b^
25 μg/mL BLPs	75.08 ± 5.30^a^	97.37 ± 0.77^a^
50 μg/mL BLPs	77.45 ± 7.73^a^	97.56 ± 0.92^a^
100 μg/mL BLPs	73.26 ± 9.24^a^	97.36 ± 1.15^a^

Note: different letters in the same column represented significant differences (*p*< 0.05).

### 3.4 BLPs inhibit glucose transport in Caco-2 cell monolayer

**FIGURE 4:**
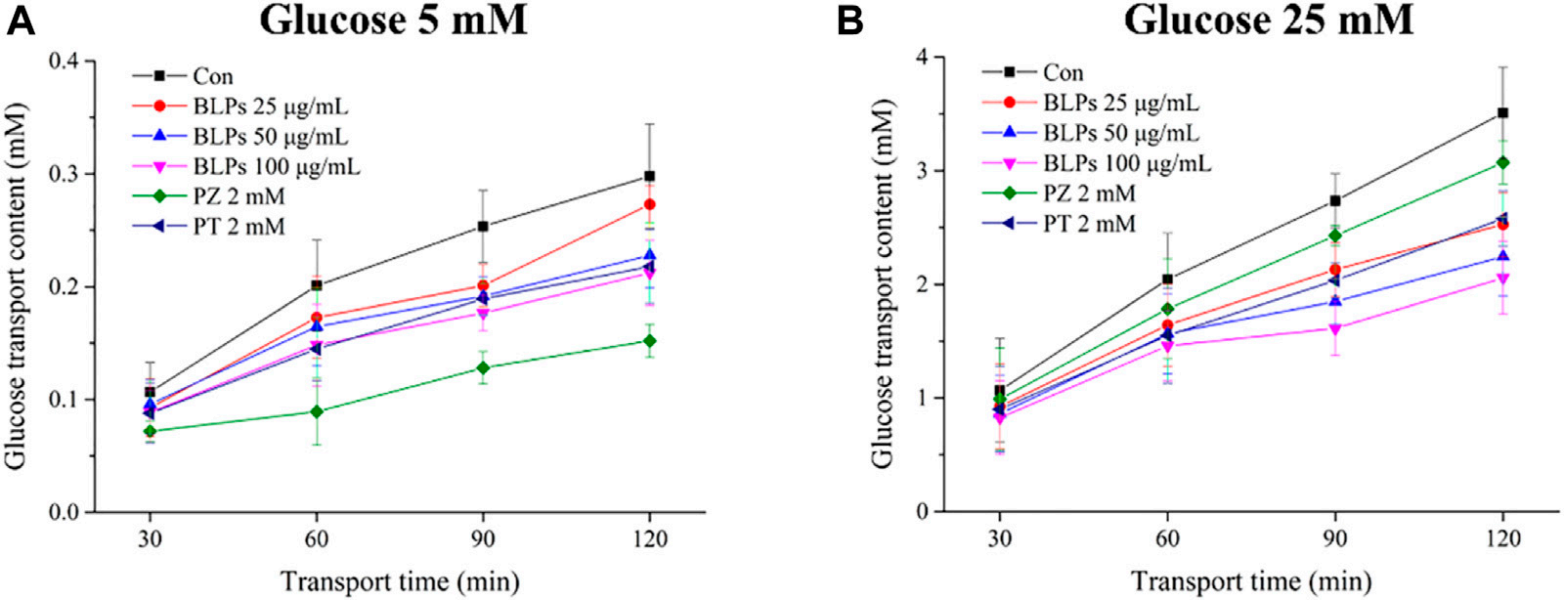
Effect of BLPs on the glucose transport content at different times (30 min, 60 min, 90 min, and 120 min) at **(A)** 5 mM low glucose condition and **(B)** 25 mM high glucose condition. Here, phloretin (PT) and phlorizin (PZ) were set as positive controls. Data are expressed as mean ± S.D. (*n* = 4). Different letters in each figure represent significant differences (*p* < 0.05).

**FIGURE 5:**
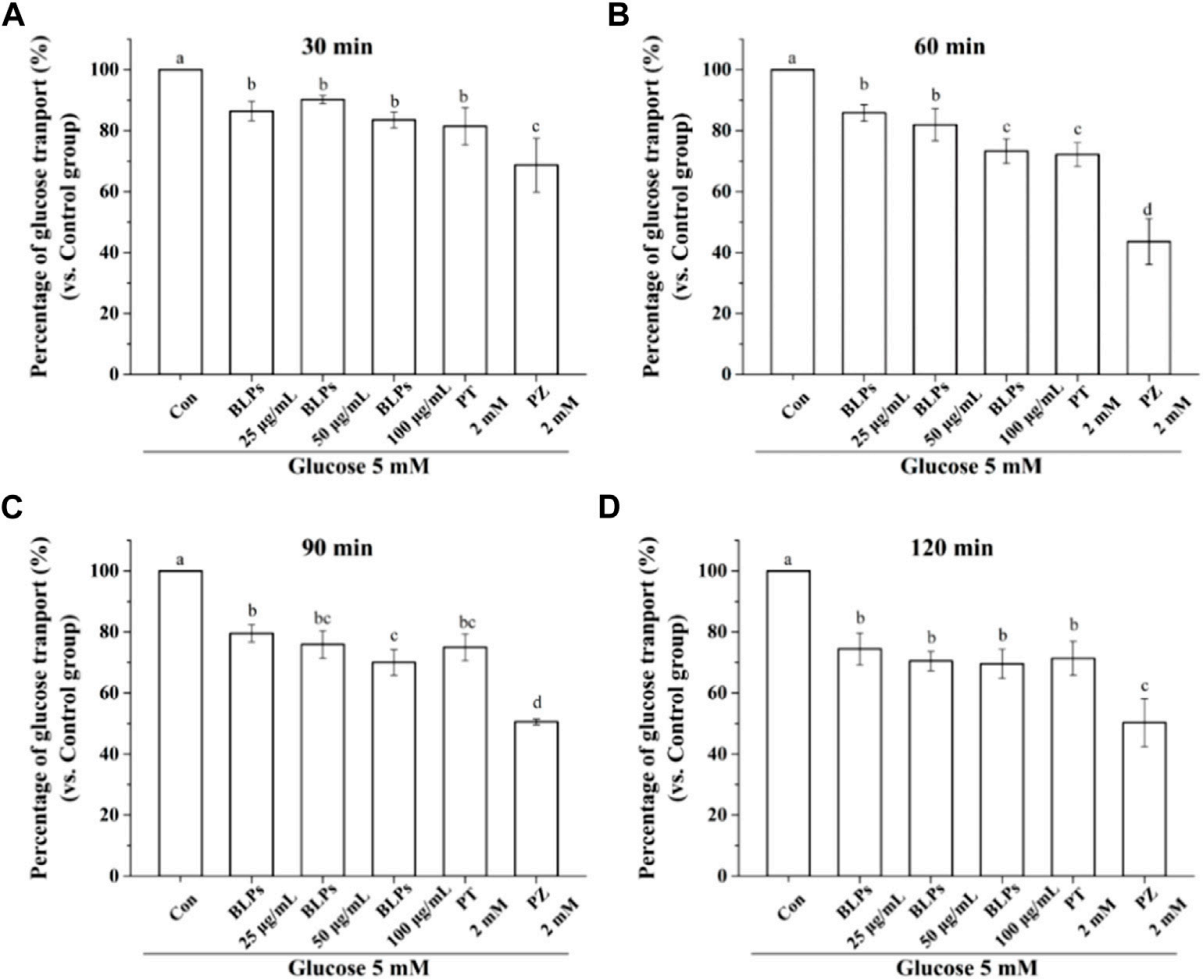
Effect of BLPs on the percentage of glucose transport at different times **(A)** 30 min, **(B)** 60 min, **(C)** 90 min, **(D)** 120 min at 5 mM low glucose condition. Here, phloretin (PT) and phlorizin (PZ) were set as positive controls. Data are expressed as mean ± S.D. (*n* = 4). Different letters in each figure represent significant differences (*p* < 0.05).

**FIGURE 6:**
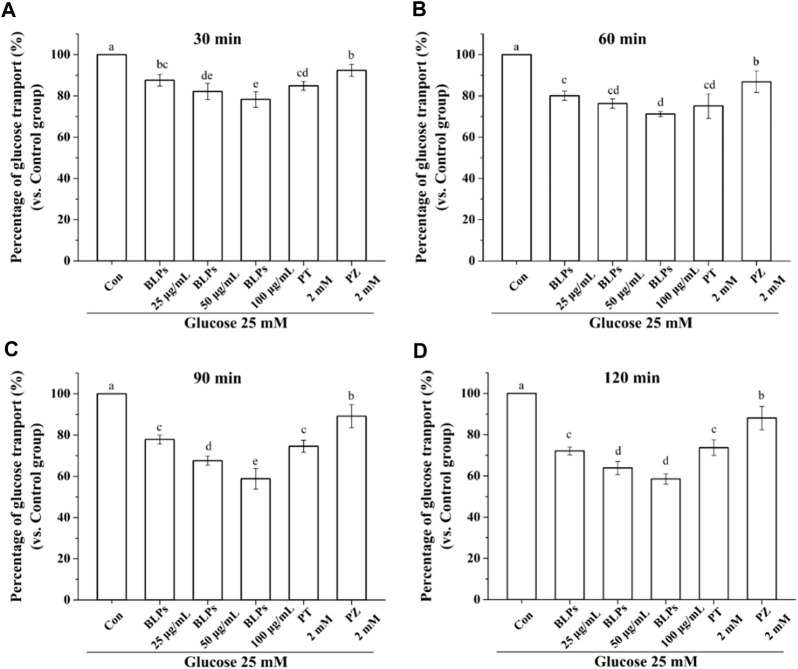
Effect of BLPs on the percentage of glucose transport at different times **(A)** 30 min, **(B)** 60 min, **(C)** 90 min and **(D)** 120 min at 25 mM high glucose condition. Here, phloretin (PT) and phlorizin (PZ) were set as positive controls. Data are expressed as mean ± S.D. (*n* = 4). Different letters in each figure represent significant differences (*p* < 0.05).

The Caco-2 cell monolayer was established to evaluate the effect of BLPs on glucose transport in the intestine. In our study, the 5 mM glucose and 25 mM glucose were used on the apical side to simulate the fasting and fed state separately. As shown in Figure 4A & Figure 5, the glucose transport percentage was decreased in the presence of BLPs under the simulated fasting condition (*p* < 0.05), which was in a time-dependent manner. In detail, 25, 50, and 100 μg/mL of BLPs had a maximum inhibition rate of 25.54%, 29.50%, and 30.41% at 120 min, respectively. It was similar to that of 2 mM phloretin with an inhibition rate of 28.60% (*p* > 0.05), but lower than that of 2 mM phlorizin (inhibition rate equal to 49.59%) (*p <* 0.05). Meanwhile, the glucose content across the Caco-2 cell monolayer was significantly decreased by BLPs in a time-dependent manner at the simulated fed state (Figure 4B; Figure 6). Comparatively speaking, BLPs can effectively inhibit the intestinal glucose transport, especially in the fed state, indicating a potential to reduce postprandial blood glucose. The hypoglycemic effect of BLPs was confirmed in the previous study (Zhang et al., 2022). As reported, the inhibitory effect of several polyphenols, such as quercetin, myricetin, caffeic acid, phloretin and phlorizin, on intestinal glucose transport was attributed to the inhibition of the expression of glucose transporters (GLUT2 and SGLT1) (Andrade et al., 2017; Pico and Martínez, 2019; Sun and Miao, 2020). We hypothesized that BLPs were the inhibitor of glucose transporters, which contributed to retarding glucose transport.

### 3.5 BLPs downregulated the expression of glucose transporters in Caco-2 cell monolayer

**FIGURE 7:**
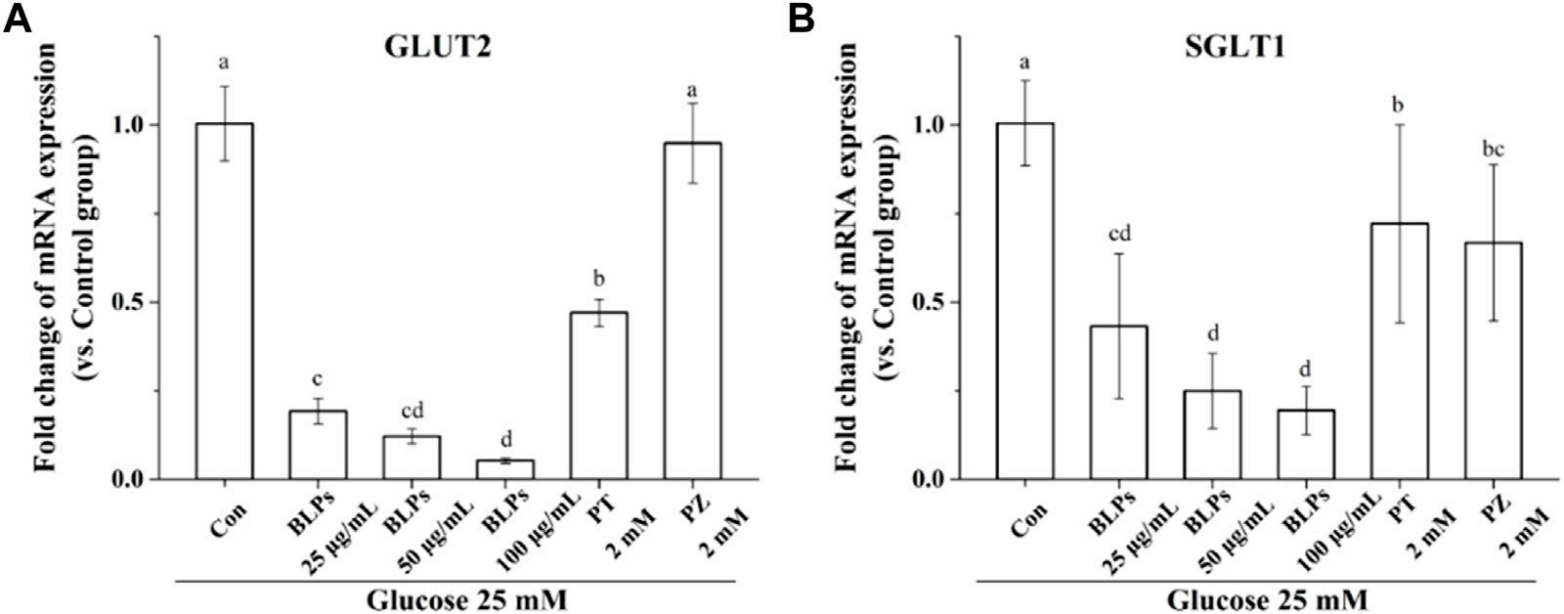
Effects of BLPs on mRNA expression levels of **(A)** *GLUT2* and **(B)** *SGLT1* in Caco-2 cell monolayer at 25 mM high glucose condition. Here, phloretin (PT) and phlorizin (PZ) were set as positive controls. Data are expressed as mean ± S.D. (*n* = 4). Different letters in each figure represent significant differences (*p* < 0.05).

**FIGURE 8:**
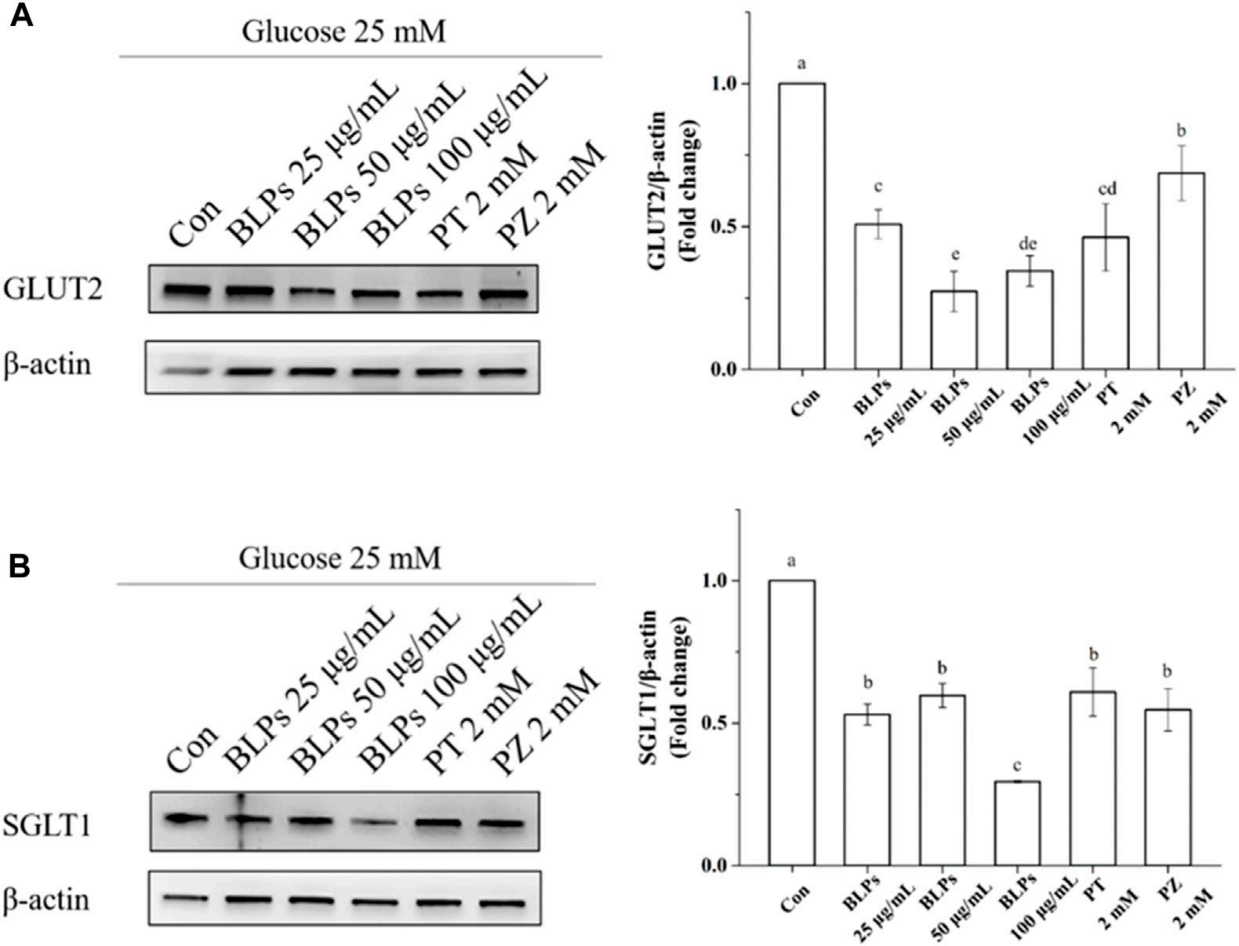
Effects of BLPs on protein expression levels of glucose transporter GLUT2 **(A)** and SGLT1 **(B)** in Caco-2 cell monolayer at 25 mM high glucose condition. Here, phloretin (PT) and phlorizin (PZ) were set as positive controls. Different letters in the figure represent significant differences (*p* < 0.05).

To verify the regulatory effect of BLPs on glucose transporters under the simulated feeding condition, the mRNA expression and protein expression of GLUT2 and SGLT1 were investigated by qPCR and western-blot analysis. As shown in Figure 7, BLPs significantly downregulated the transcript level of GLUT2 and SGLT1 in a dose-dependent manner (*p* < 0.05), which was more effective than phloretin and phlorizin (*p* < 0.05). The protein expressions of GLUT2 and SGLT1 were also significantly decreased in the presence of BLPs (*p* < 0.05) (Figure 8). The downregulating effect of BLPs at a concentration of 50 μg/mL and 100 μg/mL on GLUT2 expression level was significantly stronger than that of phloretin and phlorizin (*p* < 0.05). Meanwhile, 100 μg/mL of BLPs had a maximum inhibition on SGLT1 protein expression that was greater than that of phloretin and phlorizin (*p* < 0.05).

As reported, SGLT1 is constantly localized in the brush border membrane of the epithelial cells, and it can bind to two Na^+^ with one glucose and transport glucose to intestinal epithelial cells in a Na^+^-dependent manner. Subsequently, GLUT2 localized in the intestinal basal membrane and transports glucose into the bloodstream by facilitated diffusion. GLUT2 can also rapidly co-operate together with SGLT1 in the apical membrane of the jejunum in response to high luminal glucose concentrations (S. Liu et al., 2021). It can be seen that glucose transporters are key targets for slowing glucose absorption and transport in the intestine. In the present study, the inhibition effect of BLPs on glucose transporters is critical for reducing glucose uptake and transport.

**FIGURE 9:**
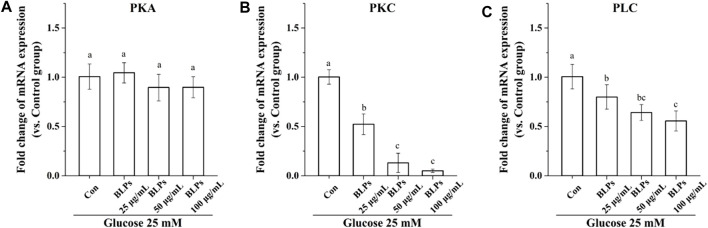
Effects of BLPs on mRNA expression levels of **(A)** *PKA*, **(B)** *PKC* and **(C)** *PLC* in Caco-2 cell monolayer at 25 mM high glucose condition. Data are expressed as mean ± S.D. (*n* = 4). Different letters in each figure represent significant differences (*p* < 0.05).

It is well-known that protein kinase A (PKA), protein kinase C (PKC) and phospholipase C (PLC) are closely related to the activity of glucose transporters GLUT2 and SGLT1 (Hameed et al., 2019). Our results showed that the mRNA expression of *PKC* and *PLC* was downregulated in the presence of BLPs (*p* < 0.05), whereas that of *PKA* was not significantly changed (*p* > 0.05) (Figure 9).

The PKA/PKC transduction pathway can regulate SGLT1 and GLUT2 activity through exocytosis and endocytosis (Torelli Hijo et al., 2019). When the PKA/PKC pathway is activated, the mRNA abundance and translation of SGLT1 and GLUT2 increase. Otherwise, PLC is essential to the activation of PKC, which contributes to the translocation of GLUT2 (Blanco et al., 2017). In the case of a healthy postprandial state, the SGLT1 is limited to escorting glucose, thereby activating the PLC in the phosphatidylinositol signaling pathway, increasing the intracellular Ca^2+^ in intestinal epithelial cells, and causing PKC activation. This led to the translocation of GLUT2 from the cytoplasm to the upper membrane of small intestinal cells, where it interacted SGLT1 in the glucose uptake and transport process (B. Li et al., 2020). However, if GLUT2 is sustainably located on the small intestinal epithelial cells, it risks the pathological increase in glucose transport that usually occurs in the postprandial state of obese and diabetic patients (Nuñez-López et al., 2013). Therefore, the above results suggested that the inhibitory effect of BLPs on SGLT1 and GLUT2 activity was beneficial in reducing the postprandial glucose response in diabetic patients. Meanwhile, the PLC-PKC pathway is involved in the inhibition of glucose transport mediated by BLPs. It was consistent with the report on cinnamon phenolic extracts, the action mechanism of which was to regulate SGK1 and the PLC/PKC pathway, leading to decreased expression of SGLT1 and GLUT2 (Y. Liu et al., 2023).

## 4 Conclusion

**FIGURE 10:**
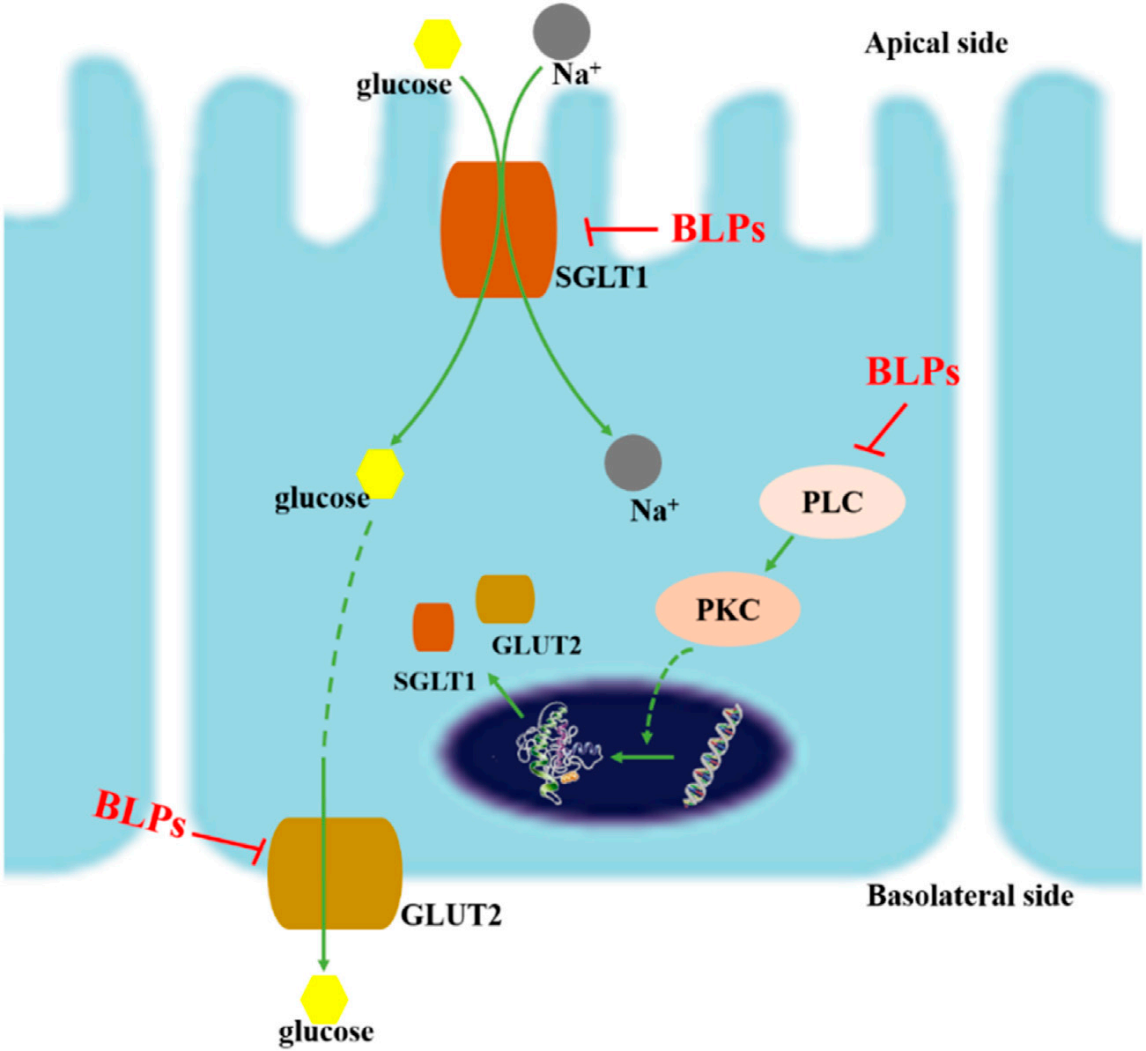
Schematic diagram of inhibition mechanism of BLPs on glucose transport. On the one hand, BLPs reduced the gene/protein expression of GLUT2 and SGLT1 *via* inhibiting the PLC-PKC pathway; on the other hand, the interaction between BLPs and GLUT2/SGLT1 changed the conformation and activity of glucose transporters, which ultimately inhibited glucose uptake and transport.

This study reported that BLPs inhibited glucose transmembrane transport by reducing the GLUT2 and SGLT1 activity in human intestinal Caco-2 cells (Figure 10). BLPs effectively retard glucose diffusion and glucose uptake. Furthermore, BLPs significantly downregulated the gene/protein expression of GLUT2 and SGLT1, as well as the transcript level of PLC and PKC. It was suggested that BLPs may alter glucose transporter activity *via* transcriptional regulation or modulation of PLC-PKC signaling cascades involved in transporter translocation. These results explained the hypoglycemic potential of BLPs *via* regulating the transcriptional expression and activity of GLUT2 and SGLT1, which is beneficial to the development of BLPs as a new functional food component. The present study provided insights for further research on targeting glucose transporters as a promising antidiabetic alternative by proanthocyanidins. However, to elucidate the in-depth mechanism of proanthocyanidins in hyperglycemia, the structure-effect relationship between proanthocyanidins and glucose transporters still needs further explanation.

## Data Availability

The original contributions presented in the study are included in the article/Supplementary Material, further inquiries can be directed to the corresponding authors.
