# Supplementary material for: Chinese bayberry (Myrica rubra Sieb. et Zucc.) leaves proanthocyanidins inhibit intestinal glucose transport in human Caco-2 cells
Source: Front Pharmacol. 2024 Mar 11;15:1284268. doi: 10.3389/fphar.2024.1284268 (PMC10961338; doi:10.3389/fphar.2024.1284268)
Supplement: Supplementary file 1 [file Table1.DOCX]

**SUPPLEMENTARY MATERIALS**

**S-METHODS AND MATERIALS**

**Extraction and purification of BLPs**

The methods of extraction and purification of BLPs were basically followed the previous method in our laboratory (Fu et al., 2014)^,^(Yang et al., 2011). Briefly, the bayberry leaves were dried in the oven at 40 °C for 12 h and grounded (<200 mesh). The leaves powder (4 kg) was extracted by soaking in 70% acetone (40 L), and the resulting aqueous phase was further extracted with hexane and dichloromethane (1:1). The obtained aqueous phase was subjected to rotary evaporation to remove the residual organic phase and then freeze-dried, which was regarded as the crude BLPs extract (CBLPs). CBLPs was then purified by a HPD-500 column to remove sugar by water and eluted with 70% ethanol. The eluate was evaporated to freeze-dried yielding a brown powder, which was regarded as the resin purified BLPs (RPBLPs). Afterwards, RPBLPs was purified by a Sephadex LH-20 (300 mm × 30 mm i.d.) column to remove most of the flavonoids by 90% methanol and then eluted with 50% acetone. The eluate was subjected to rotary evaporation to remove the residual organic phase and then freeze-dried to obtain the final product of BLPs.

**Determination of PAs content.**

The content of BLPs was determined using vanillin assay (Sun et al., 1998). Briefly, 200 μL of BLPs solutions (0.5 mg/mL) was mixed with 500 μL of vanillin-methanol (1%, w/v) and 500 μL of H_2_SO_4_-methanol (20%, v/v) to state reaction under water bath at 30 °C for 15 min. The absorbance at 500 nm was determined using a microplate reader. Methanol was used as the blank. The BLPs content was expressed as EGCG equivalent.

**Thiolysis and HPLC analysis of BLPs.**

Thiolysis of BLPs and HPLC analysis were according to the methods of Chi Gao’s (Gao et al., 2018) with some modification. Firstly, BLPs were prepared a 10 mg/mL solution dissolving in 95% ethanol, and then depolymerized using 500 mg/mL cysteamine-95% ethanol with 0.3 M HCl at 70℃ for 20 min. The solution of thiolysis product was filtered through a filter membrane (0.45 μm) and analyzed by Agilent 2695/2998 HPLC-DAD system (California, United States). The separation and detection conditions were as follows: a GU-C18M column (250 × 4.6 mm, 5 μm, Green mall, Jiangsu, China), column temperature at 25 ℃, detection wavelength at 280 nm, mobile phase A of water (2% acetic acid), and mobile phase B of methanol, flow rate with 1 mL/min. The gradient elution condition: 10% B (0-31 min), 10-30% B (31-40 min), 30-80% B (40-43.6 min), and 80-10% B (43.6-45 min), followed by a 10 min equilibration. The mean degree of polymerization (mDP) was calculated according to the corresponding peak areas of thiolysis products.

**Characterization of BLPs.**

The characterization of BLPs and its structural elucidation have been previously reported by our group (Fu et al., 2014)^,^(Yang et al., 2011). Yang et al. (Yang et al., 2011) found that the structure of BLPs was particular, which was of the prodelphinidin type. By the acid catalysis with excess phloroglucinol, epigallocatechin-3-O-gallate (EGCG) and traces of epigallocatechin (EGC) were detected as the extension units, but only EGCG was present in the terminal units. BLPs exhibited a 2,3-cis configuration, and more than 98% of them were galloylated. Afterwards, Fu et al. (Fu et al., 2014) further supported the results of Yang et al. (Yang et al., 2011). And the fractions from BLPs and their identification from normal-phrase HPLC-ESI/MS and reverse-phase-ESI/MS were also reported (Table S2). The major components of BLPs were: 5.4% monomers, 10.9% dimers, 13% trimers, 47.8% tetramers, 22.9% polymers and other polyphenols.


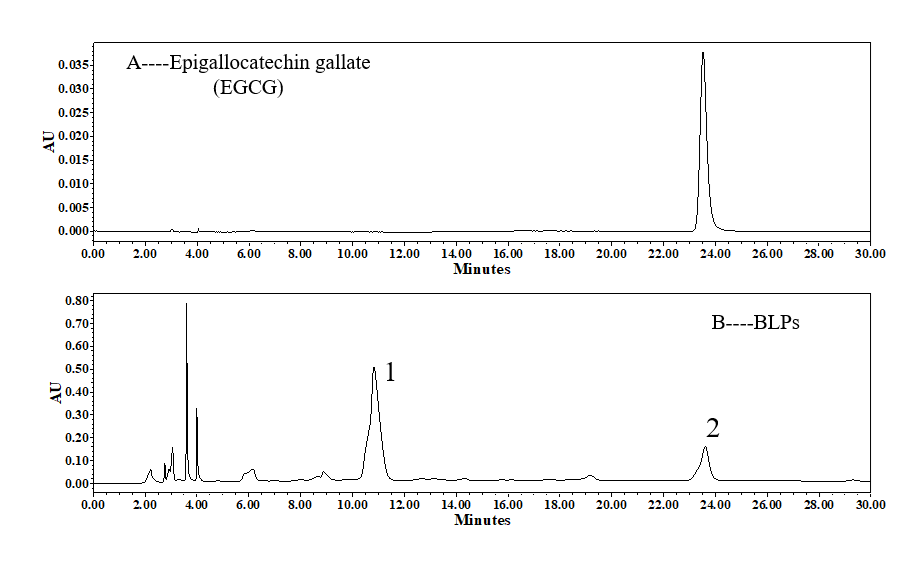


**Fig. S1.** HPLC chromatograms (detected at 280 nm) of standards of EGCG (A) and thiolyzed BLPs (B). Peak 1: EGCG cysteamine thioether (thio-EGCG); peak 2: EGCG

**Table S1.** Mean degree of polymerization (mDP) and content of proanthocyanidins (PAs) of BLPs.

| Sample | PAs (% EGCG equivalent) | mDP |
| --- | --- | --- |
| BLPs | 85.7±1.1 | 7.3 |

**Table S2.** Fractions from BLPs and their identification from normal-phase preparative HPLC-ESI/MS and reverse-phase HPLC-ESI/MS.

| Fractions^a^ | Yield^b^ (mg/200mg) | MW^c^ | Tentative identification^d^ |
| --- | --- | --- | --- |
| 1 | 10.8±0.3 | 616 | myricetin deoxyhexoside-gallate |
| 2 | 4.6±0.1 | 744, 882 | (E)GC+(E)CG, 2(E)CG, 2(E)C+(E)GC |
| 3 | 8.6±0.3 | 762 | (E)GC+(E)GCG |
| 4 | 8.6±0.4 | 914 | 2(E)GCG |
| 5 | 4.3±0.2 | 1066 | 2(E)GC+(E)GCG |
| 6 | 8.7±0.2 | 1218 | (E)GC+2(E)GCG |
| 7 | 13.0±1.5 | 1371 | 3(E)GCG, 3(E)GC+(E)GCG |
| 8 | 19.6±0.6 | 1523 | 2(E)GC+2(E)GCG |
| 9 | 32.2±1.6 | 1675 | (E)GC+3(E)GCG |
| 10 | 43.8±1.0 | 1827 | 3(E)CG+(E)GCG, 4(E)GCG |

^a^ Fractions, Fractions and their identification from normal-phase preparative HPLC- ESI/MS and reverse-phase HPLC-ESI/MS.

^b^ Yield, yield of one injection of preparative HPLC, that is, milligram per 200 milligrams BLPs.

^c^ MW, molecular weight.

^d^ Tentative identification, (E)GC, (E)GCG, (E)CG are abbreviations for (epi)gallocatechin, (epi)gallocatechin-3-O-gallate, (epi)catechin-3-O-gallate.


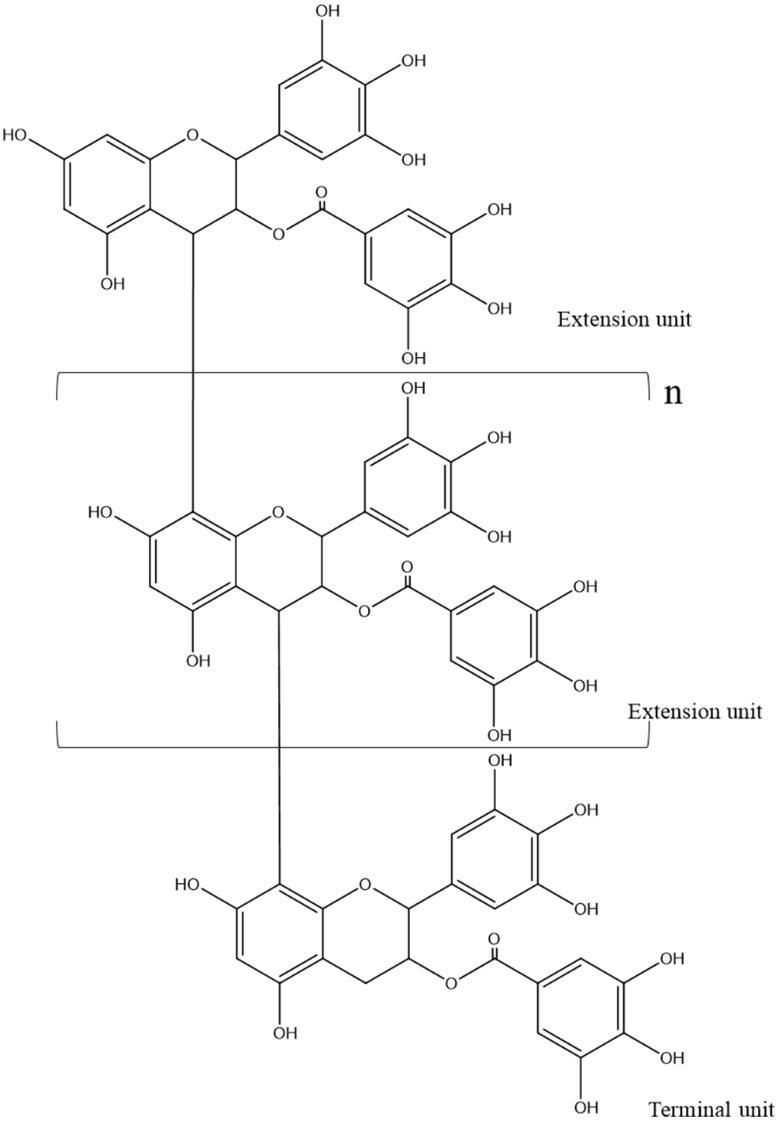


**Fig. S2.** Structural representation of BLPs molecules, with the EGCG as extension unit and terminal unit.

**S-REFERENCES:**

Fu, Y., Qiao, L., Cao, Y., Zhou, X., Liu, Y., & Ye, X. (2014). Structural elucidation and antioxidant activities of proanthocyanidins from Chinese bayberry (Myrica rubra Sieb. et Zucc.) leaves. *PLoS ONE*, *9*(5), e96162. https://doi.org/10.1371/journal.pone.0096162

Gao, C., Cunningham, D. G., Liu, H., Khoo, C., & Gu, L. (2018). Development of a thiolysis HPLC method for the analysis of procyanidins in cranberry products. *Journal of Agricultural and Food Chemistry*, *66*(9), 2159–2167. https://doi.org/10.1021/acs.jafc.7b04625

Sun, B., Leandro, C., Ricardo Da Silva, J. M., & Spranger, I. (1998). Separation of grape and wine proanthocyanidins according to their degree of polymerization. *Journal of Agricultural and Food Chemistry*, *46*(4), 1390–1396. https://doi.org/10.1021/jf970753d

Yang, H., Ye, X., Liu, D., Chen, J., Zhang, J., Shen, Y., & Yu, D. (2011). Characterization of unusual proanthocyanidins in leaves of bayberry (Myrica rubra Sieb. et Zucc.). *Journal of Agricultural and Food Chemistry*, *59*(5), 1622–1629. https://doi.org/10.1021/jf103918v
